# Development of Biological Movement Recognition by Interaction between Active Basis Model and Fuzzy Optical Flow Division

**DOI:** 10.1155/2014/238234

**Published:** 2014-04-30

**Authors:** Bardia Yousefi, Chu Kiong Loo

**Affiliations:** Department of Artificial Intelligence, Faculty of Computer Science and Information Technology, University of Malaya, 50603 Kuala Lumpur, Malaysia

## Abstract

Following the study on computational neuroscience through functional magnetic resonance imaging claimed that human action recognition in the brain of mammalian pursues two separated streams, that is, dorsal and ventral streams. It follows up by two pathways in the bioinspired model, which are specialized for motion and form information analysis (Giese and Poggio 2003). Active basis model is used to form information which is different from orientations and scales of Gabor wavelets to form a dictionary regarding object recognition (human). Also biologically movement optic-flow patterns utilized. As motion information guides share sketch algorithm in form pathway for adjustment plus it helps to prevent wrong recognition. A synergetic neural network is utilized to generate prototype templates, representing general characteristic form of every class. Having predefined templates, classifying performs based on multitemplate matching. As every human action has one action prototype, there are some overlapping and consistency among these templates. Using fuzzy optical flow division scoring can prevent motivation for misrecognition. We successfully apply proposed model on the human action video obtained from KTH human action database. Proposed approach follows the interaction between dorsal and ventral processing streams in the original model of the biological movement recognition. The attained results indicate promising outcome and improvement in robustness using proposed approach.

## 1. Introduction

Human action recognition in monocular video is one of the important subjects regarding video applications, for example, human computer interaction, video search, and so forth. It has been researched in different fields such as neurophysiology, psychophysics, and experimentations on imaging, and some areas in cortical engaged in it have been acknowledged. In general, human action recognition in the video stream using video processing and such methods in the proposed area is almost categorized by two techniques: one of them by using the global feature extraction form video streams tries to allocate a particular label to the whole video. The mentioned technique clearly needs unchanged observer within the video and considers the environments where actions occur [1]. Second technique considers local features regarding every frame and considers the label for distinct action. Afterward, by mechanisms of simple voting for global label regarding sequence can be attained. Temporal analysis for getting the features in every frame and classification is based on the observation in temporal window. It is important that both of these approaches have attained significant outcomes in such area (see [2]). One of the important factors of the complex action recognition and discriminating among unlike human motion styles and different individuals is learning [3] and also it is fundamental in recognition of 3D stationary human motion [4]. The human action recognition using frames of the video can be categorized as object recognition problem. It is supposed to handle the object variations (e.g., style, size, etc.). Meanwhile human brain is able to excellently categorize human object in different classes of action; recent methods are inspired by biological outcomes of computational neuroscience [5, 6]. In the primary visual cortex (V1), procedure of images is more sensitive on barlike structures. Responses of V1 are combined together by extrastriate visual areas and passed to inferotemporal cortex (IT) for tasks of recognition [7].

## 2. Biologically Inspired Model

We follow the model of biological movement based on four assumptions, which are reliable by physiological and anatomical information [8]. The model splits into two corresponding preprocessing streams [9–12] parallel to dorsal and ventral streams which are specified for analysis of optic-flow and structure information, respectively. The model has used neural feature detector for extraction of optical flow and form features hierarchically considering size and style independency for both pathways; here we use synergetic neural network in both feedforward pathways for extraction of the structure and optical flow information. The corresponding results on the stationary human motion recognition reveal that discrimination can be accomplished through particularly small latencies, constructing an important role of top-down signals unlikely [9]. The motion is shown based on a set of patterns which are learned. The body shapes are determined by mentioned patterns like sequences of snapshots. The pathway regarding structure of body made by neurons and the complex patterns of motion pathway has been presented applying optical flow. This statement is a fundamental hypothesis of our model. The proposed model expands an earlier model used for the stationary objects [2, 7, 8, 12, 13] recognition by adding and combining the information over time in the dorsal and ventral pathway. Some visual physiologists have the regular belief regarding the proposed model [8]. It can be a good pertaining to quantity tool for organizing, summarizing, and interpreting existent information. The initial structure design is based on the data provided by neurophysiological and physiological evidences. This developed structure implements the quantitative estimation through computer simulations. Motion recognition and visual data have been involved in the model architecture. The proposed model has two separated pathways regarding form and motion information analysis. The information of two processing streams cooperates at few levels in the mammalian brains [14, 15]. Mentioned coupling is able to ease the model integration, for instance, in STS level [16] and it develops the performance of recognition without varying the fundamental results. Both pathways made up a hierarchy of neural feature detectors, which they use here for getting predefined templates regarding motion and form of the movement and make selective recognition.

### 2.1. Form Pathway

In the biological motion model, form pathway considers the system work as object recognition task and more involves in recognition of human body shape through sequential snapshots by camera. In general, few models have been proposed which are plausible and neurophysiological about recognizing stationary form (e.g., [12]). Our proposed form pathway model follows an object recognition model [12] which is composed of form features detectors. The proposed approach has capability to be reliable like data obtained from neurophysiological information concerning scale, position, and sizes invariance which need further computational load along hierarchy. For modeling the cells in the primary visual cortex (V1) initial level of the structure pathway comprising detectors of local direction has been organized. Techniques having Gabor-like filters for modeling the detectors have good constancy by simple cells [17]. Two spatial scales by 2 factors for its differentiation and eight directions have been considered for the orientation detectors model. The neurons in monkey V1 range can influence the sizes of perceptive field in the receptive fields [18]. The scale and location detectors are located in the following level of this pathway that finds the information of local direction. Moreover, there is an approximated independency for scales and spatial location inside receptive fields. Perhaps, the complex-like cells in V1 area or in V2 and V4 are invariant regarding position varying responses (see [8]) and size independence is typical in area V4. These two areas (V2 and V4) are more selective for difficult form features, for example, junctions and corners whereas they are not suitable for recognition of the motion. To have an independent scale and position using mechanism of neurophysiologically through plausible model choosing the detectors responses by different directions and receptive field scales and locations. The pooling achieved through using maximum similarity operation as it mentioned in [19] some complex cells in cats visual cortex and areas V4 of macaques [20] reveal a maximum computing behavior. Afterward, the snapshots detectors are used for finding shapes of the human body model similar to area IT (inferotemporal cortex) of the monkey where the view-tuned neurons are located and model of complex shapes is tuned [21]. Snapshot neurons are similar to view-tuned neurons in area IT that give independent scale and position. Previous models used Gaussian Radial Basis functions for modeling and it adjusts during training. Then, the optimum set of training frames was analyzed and the learning rate was estimated (see [8, 12]). Neurons regarding motion pattern which are located in the form pathway highest level integrate the outcomes of snapshot neurons. Following presented biologically inspired methods regarding object recognition and its applications, we develop neurobiological model [2, 8, 22] of processing of the shape and motion in the dorsal stream in the visual cortex using active basis model as computational mechanisms into the feedforward aligned with motion pathway (optical flow).

### 2.2. Motion Pathway

In the motion pathway biological movements were recognized by using patterns of optical flow. The optical flow finds out the movement pattern which has consistency with neurophysiological information from hierarchy of neural detectors. In areas MT and V1 there are some neurons for motion and direction selection in first level of motion pathway, respectively. There are many models for local motion estimation which are neurophysiologically plausible; we directly compute the response of motion-selective neurons and optical flow. Location form of the movement is analyzed in the second level of motion pathway. Local detector of optical flow is connected with motion patterns and the model comprises population of four directed neurons in area of MT. Also, motion edges selectors are sensitive in two opposite direction that find in areas of MT, MSTd, MSTl, and many parts of the dorsal steams and probably in the kinetic occipital area (KO) [8]. The proposed model and object specific motion position will be obtained by maximum pooling from motion position detector and considering motion selective edges which can be like MT [18] and MSTl [23] in macaque monkey. There is similarity between form pathway and third level motion pathway by snapshots neurons regarding model prediction. Motion pathway collects the optical flow patterns neurons outcome and smooths them to model them similarly with form pathway. The proposed approach active basis model [24] using spatiotemporal features intermediate complexity simulates the form pathway and optical flow [25] represents motion pathway for stimulating the proceeded areas MST and MT in dorsal stream where neurons have significant location and unchangeable scale. The obtained information will be processed by synergetic neural network [7, 13] and also predefined prototype which will be descried later. These patterns attain prototypes outcomes from active basis model and optical flow. Applying proposed approach is a simulation of both pathways in the primary visual cortex (V1) and projection of vertical stream in areas V2 and V4; see [8].

## 3. System Overview

The proposed system addresses a hierarchy of feature detectors which helps the system to be biologically inspired. Like [4, 8] and based on [8], we consider a basic consideration mechanism: our input is images obtained from video sequences and they have fixed size. The model is not sensitive regarding the background information.

### 3.1. Active Basis Model for Form Pathway

Active basis model [2] applying Gabor wavelets (for elements dictionary) offers deformable biological template; however, Gabor filters and Histograms of Oriented Gradients are presented for finding the human object. Shared sketch algorithm (SSA) followed through Ada-boost (like [26]). In every iteration, SSA following matching pursuit chooses an element of wavelet. It checks the objects number in different orientation, location, and scale. Selecting the small number of elements from the dictionary for every image (Sparse coding), therefore there can be representation of image using linear combination of mentioned elements by considering U as a minor residual. Consider

(1) $$\begin{array}{c} I=\overset{n}{\underset{i=1}{\sum }}c_{i}\beta_{i}+\epsilon , \end{array}$$

where *β* = (*β*_*i*_, *i* = 1,…, *n*) is set of Gabor wavelet elements and components of sin and cosine, *c*_*i*_ = 〈*I*, *β*_*i*_〉, and *ϵ* is unsolved image coefficient [24]. By using wavelet sparse coding the large number of pixels reduces to small number of wavelet elements. Sparse coding can train natural patches of image to a Gabor-like wavelet elements dictionary which carries the simple cells in V1 properties [6, 24]. The extraction of local shapes will be separately done for every frame like [2] which computes the responses of filter orientation and density for every pixels. Also, the active basis model [24] uses the Gabor filter bank but in different form (see Figure 2). A Gabor wavelets dictionary, comprising *n* directions and *m* scales, is in the form of *GW*_*j*_(*θ*, *ω*), *j* = 1,…, *m* × *n* where *θ* ∈ {*kπ*/*n*, *k* = 0,…, *n* − 1} and $\omega =\{\sqrt{2}/i$, *i* = 1,…, *m*}. Gabor wavelet features signify the object form as a small variance in size and location and posture. The shape structure considers being safe maintained throughout the procedure. Response (convolution) to each element offers form information with *θ* and *ω*. Consider

(2) B=〈GW,I〉=∑∑GW(x0−x,y0−y:ω0,θ0)I(x,y).

Let *GW*_*j*_ be a  [*x*_*g*_, *y*_*g*_], let *I* be [*x*_*i*_, *y*_*i*_] matrices, and let response of *I* to *GW* be a [*x*_*i*_ + *x*_*g*_, *y*_*i*_ + *y*_*g*_]. Therefore, previous convolution of both matrices must be padded through sufficient zeros. Consequence of convolution can be eliminated via cropping the result. Additional technique would be to shift back the center of the frequencies (zero frequency) to center of the image though it might reason for loosing data. Obtaining training image set {*I* ^*m*^, *m* = 1,…, *M*}, the joint sketch algorithm consecutively chooses *B*_*i*_. The fundamental opinion is to find *B*_*i*_ so that its edge segments obtained from *I*_*m*_ become maximum [24]. Afterward, it is necessary to compute [*I* ^*m*^ · *β*] = *ψ*|〈*I* ^*m*^·*β*〉|^2^ for different *i* where *β* ∈ *Dictionary* and *ψ* represents sigmoid, whitening, and thresholding transformations and then maximizing [*I* ^*m*^ · *β*] for all possible *β* will be computed. Let *β* = (*β*_*i*_, *i* = 1,…, *n*) be the template for every training image *I* ^*m*^; scoring will be based on

(3) $$\begin{array}{c} M\left(I^{m},\theta \right)=\overset{n}{\underset{i=1}{\sum }}\delta_{i}\left|I^{m},\beta \right|-\log\Phi \left(\lambda \delta_{i}\right). \end{array}$$

*M* is the match scoring function and *δ*_*i*_ is obtained from ∑_*n*=1_^*M*^[*I* ^*m*^, *β*] regarding steps selection and Φ is nonlinear function. The logarithmic likelihood relation of exponential model attains from the score of template matching. Vectors of the weight are calculated by maximum likelihood technique and are revealed by Δ = (*δ*_*i*_, *i* = 1,…, *n*) [24]. Consider

(4) $$\begin{array}{c} \mathrm{Max}\left(x,y\right)=\text{ma}{\text{x}}_{(x,y)\in D}M\left(I_{m},\beta \right). \end{array}$$

* Max*⁡(*x*, *y*) calculates the maximum matching score obtained previously. *D* represents the lattice of *I*. Here, there is no summation because of updating the size based on training system on frame (*t* − 1). Moreover, the method tracks the object applying motion feature for getting displacement of moving object.

### 3.2. Optical Flow for Motion Pathway

Using optical flow (as it is aforementioned) is one of the effective methods in human action recognition (see [27]). For having the features regarding motion of subject, layer-wise optical flow estimation has been done. A mask which reveals each layer's visibility is the main difference between estimation of traditional and layer-wise optical flow. The mask shape is able to perform fractal and arbitrary considering match will occur inside mask in the pixel-wised form (see [25]). We use the layer-wise optical flow method in [25] which has baseline optical flow algorithm of [28–30]. *M*_1_ and *M*_2_ are visible masks for two frames *I*_1_(*t*) and *I*_2_(*t* − 1), the field of flow from *I*_1_ to *I*_2_ and *I*_2_ to *I*_1_ is represented by (*u*_1_, *v*_1_) and (*u*_2_, *v*_2_). The following terms will be considered for layer-wise optical flow estimation. Objective function consists of summing three parts; visible layer masks match to these two images using Gaussian filter which called data term matching *E*_*γ*_^(*i*)^, symmetric *E*_*δ*_^(*i*)^, and smoothness *E*_*μ*_^(*i*)^. Consider

(5) $$\begin{array}{c} E\left(u_{1},v_{1},u_{2},v_{2}\right)=\overset{2}{\underset{i=1}{\sum }}E_{\gamma }^{\left(i\right)}+\rho E_{\delta }^{\left(i\right)}+\xi E_{\mu }^{\left(i\right)}. \end{array}$$

After optimization of objective function and using outer and inner fixed-point iterations, image warping, and coarse to fine search, we attain flow for both bidirections. Compressed optic flow for all the frames is calculated by straight matching of template to the earlier frame by applying the summation of absolute difference (*L*1 − norm⁡). Though optic flow is particularly noisy, no smoothing techniques have been done on it as the field of flow will be blurred in gaps and specially the places where information of motion is significant [22]. To obtain the proper response of the optical flow regarding its application in the proposed model, optical flow will be applied for adjusting the active basis model and making it more efficient. To achieve a representation reliable through the form pathway, the optic flow estimates the velocity and flow direction. The response of the filter based on local matching of velocity and direction will be maximal as these two parameters are continuously changing.

### 3.3. Synergetic Neural Network Classifier

Analyzing the human brain cognitive processes [31, 32], particularly the visual analysis, we apprehend that the brain is persistently involved in a big amount of the perception reprocessing, subconscious mind, filtering, decomposition, and synthesis. The brain of human is a cooperative system; in some cases, cognitive processes can be supposed to depend on the self-organizing pattern formation. Based on this knowledge, Haken presents synergetic neural network as one pattern recognition process which performs in the brain of the human. A joint method for association of trained samples is the values of feature averaging (see [33]). He revealed a collaborative pattern recognition of a top-down thinking: pattern recognition process can be comprehended like a specific order parameter competition process for recognition mode *q* can construct a dynamic process, so *q* after middle state *q*(*t*) into a prototype pattern *v*_*k*_. Though it is not flexible enough for direction changing, therefore the boundaries of these templates are not clear. Applying learning object in the same view is a technique for dealing with inflexibility which will limit the task of classification. Algorithm of melting is introduced by [3] for objects combination in diverse pose. Assume a trained object sample Í_*i*_ contains *n* pixel values. By reshaping Í_*i*_ to *v*_*i*_ which is a column vector matrix and normalization we will have

(6) $$\begin{array}{c} \overset{n}{\underset{j=1}{\sum }}v_{i}j=0,\;\;\overset{n}{\underset{j=1}{\sum }}v_{ij}^{2}=1, \end{array}$$

where *q* is the input mode and *q*_0_ is the initial values of the state vector for attention parameters, which we will discuss later. Connected prototype matrix *V* ^+^ calculates *V* ^+^ = (*V* ^+^ *V*)*V*(1). Let *V* be all the learn samples set *v*_*i*_ = 1,…, *m*. and every column satisfies condition of orthonormal *v*_*k*_^+^ *v*_*j*_ = *δ*_*ij*_, for all *j* and *k*, where *δ*_*ij*_ is delta of Kronecker. For a sample examination *q*, parameters of order signify test sampling matching. Class parameter of order for *k* derives as *ϵ*_*k*_ = *v*_*k*_^+^, *k* = 1,…, *m*. Due to pseudoinverse overfitting, sometimes melting fails to generalize the learning. A penalty function is presented as Most Probable Optimum Design (MPOD) to improve the generalization and classify face object pose application (see [34]). Following this modification, the melting combination of similar object patterns into a template is useful for classification. So synergetic template is

(7) $$\begin{array}{c} v_{p}^{+}=E{\left(V^{T}V+P_{1}O+P_{2}I\right)}^{-1}V^{T}. \end{array}$$

*I*, *O*, *P*_1_, and *P*_2_ are identity matrix, unitary matrix, and coefficients of penalty. *E* is an enhanced identity matrix; every element of *E* is a row vector of size *j* as the following:

(8) $$\begin{array}{c} E=\left[\begin{array}{cccc} e_{1}^{n(1)} & e_{0}^{n(2)} & \cdots & e_{0}^{n(M)}\\ e_{0}^{n(1)} & e_{1}^{n(2)} & \cdots & e_{0}^{n(M)}\\ \cdots & \vdots & \ddots & e_{0}^{n(M)}\\ e_{0}^{n(1)} & e_{0}^{n(2)} & \cdots & e_{1}^{n(M)} \end{array}\right],\\ e_{0}^{i}=\left(0,\ldots ,0\right),\;\;e_{1}^{i}=\left(1,\ldots ,1\right). \end{array}$$

It can be a relevant feedback and self self-attentive for similarity measurement. But here the proposed model uses synergetic neural network two times, once for making the templates in each pathway and in the second time in the final classification.

### 3.4. Fuzzy Optical Flow Division

Fuzzy logic is a logic having multivalued that is originated from theory of fuzzy set found by Zadeh [35] and it deals with reasoning approximation [35]. It provides high level framework targeted at approximation reasoning which can appropriately deliver the imprecision and uncertainty together in linguistic semantics and model expert heuristics and handles requisite high level organizing principles. Fuzzy logic can be an important balancing method which is plausible and justifies combining approaches together for designing the classification, decision, and inference systems [36]. Various fuzzy inference systems have been proposed and have many applications through Max-Min fuzzy operations. However, Leotamonphong and Fang mentioned that composition of max-min is suitable only when a system allows no compensation among the elements of a solution vector [37]. A time dependent fuzzy system also has been used many times regarding solution of control and classification and so forth. Chen and Liu [38] present a delay-dependent robust fuzzy control for a class of nonlinear delay systems via state feedback [38].* Problem statement and preliminary:* after applying optical flow, the velocity of human object will be considered for both *x* and *y* directions. In general, *v*_*x*_, *v*_*y*_ ∈ *R* ^*m*×*n*^ where *m* and *n* are sizes of image frame from input video stream.

(1)  *μ*_*v*_*x*__^*C*_1,2_^(*x*), *μ*_*v*_*x*__^*C*_2,4_^(*x*), *μ*_*v*_*x*__^*C*_1,2_^(*y*), and *μ*_*v*_*x*__^*C*_2,4_^(*y*) are triangular membership functions for *v*_*x*_ and it will be the same for *v*_*y*_ velocity in *x* and *y* directions these represent the quaternion correlator for outputs of motion pathway. The fuzzification is done through triangular membership function as activation functions

(9) $$\begin{array}{c} \mu_{v_{x}}^{C_{1,2}}\left(x\right)=\{\begin{array}{cc} \frac{x}{C_{1,3}} & 0<x\leq C_{1,3}\\ \frac{C_{2,4}-x}{C_{1,2}-C_{3,4}} & C_{1,3}<y\leq C_{2,4}, \end{array}\\ \mu_{v_{x}}^{C_{2,4}}\left(x\right)=\{\begin{array}{cc} \frac{x-C_{1,3}}{C_{2,4}-C_{1,3}} & C_{1,3}<x\leq C_{2,4}\\ \frac{C_{1,3}-x}{C_{1,3}-C_{2,4}} & C_{2,4}<x\leq n, \end{array}\\ \mu_{v_{x}}^{C_{2,4}}\left(y\right)=\{\begin{array}{cc} \frac{y}{C_{3,4}} & 0<y\leq C_{3,4}\\ \frac{C_{1,2}-y}{C_{1,2}-C_{3,4}} & C_{3,4}<y\leq C_{1,2}, \end{array}\\ \mu_{v_{x}}^{C_{1,4}}\left(y\right)=\{\begin{array}{cc} \frac{y-C_{3,4}}{C_{1,2}-C_{3,4}} & C_{3,4}<x\leq C_{1,2}\\ \frac{m-y}{m-C_{1,2}} & C_{1,2}<y\leq m. \end{array} \end{array}$$

The position of the highest velocity in *x*,*y* estimated by evaluating the amount of membership functions and then membership function related to every cell will be based on aggregating *x*,*y* for each velocity. It will evaluate both cases of velocities. *μ*_*v*_*x*__^*C*_*i*_^ and *μ*_*v*_*y*__^*C*_*i*_^ are showing the membership in each cell where *z* is number of the cell (*i* = 1,…, 4). Consider

(10) $$\begin{array}{c} \mu_{v_{x}}^{C_{i}}=\max\left\{\mu_{v_{x}}^{C_{i}},\mu_{v_{y}}^{C_{i}}\right\}. \end{array}$$

(2) Determine the value of motion information in motion pathway in frame time **t**. As information of velocities can be unstable due to shaking the camera or different style in human object meanwhile he is acting in front of camera, the amount of velocity is dependent on time. The definition of time in this context is based on the frame time per second. Here, this dependency implements by considering the previous frame membership value. Proposed time dependent fuzzy optical flow division can be utilized for representing a class of optical flow divisions with fuzzy inference rules concerning time regarding every frame of video stream as unit of the time is defined here, as follows:

(11) μ~vCi(t)=μ~vCi(t−τ)+ηvCi(t)(1−μ~vCi(t−τ))t∈[t0,t0+kτ], k∈(0,1,…,N)

where *τ* is the frame time which is a parameter for camera and *k* is numbers of frames pasted from the cell changing (it means *k* will be reset after varying of the cell membership). *N* is the maximum number of frame distance from present frame which does not unreasonably increase membership function value. We call *η*_*v*_^*C*_*i*_^(*t*) memory coefficient function and add to the membership function of the winner cell and define as follows:

(12) ηvCi(t)={1k+βμvCj(t)≤μ~vCj(t=τ)−1k+β′μvCj(t)>μ~vCj(t=τ),k≥0, k≠−β, k≠−β′

Let *β* as adjustment parameter be tuned in the system. *C*_*j*_ presents the cell which is different from *C*_*j*_ and has maximum velocity among all cells in optical flow division. *t* is the time of frame where one division of the optical flow has the highest membership amount as compared with other divisions and it will be restarted by changing the division.

(3) Gather values produced in previous memberships in every optical flow division in each frame by the following rules.

(a) Flow of upper limb is attained by association of optical flow fuzzy amounts for *C*_1_ and *C*_2_. Membership value reveals the flow for upper limb of human object. It is mentioned as follows:
  (13) $$\begin{array}{c} \mu_{\text{Upper-Limb}}\left(t\right)={\tilde{\mu }}_{v}^{C_{1}\cup C_{2}}\left(t\right)=\max\left\{{\tilde{\mu }}_{v}^{C_{1}}\left(t\right),{\tilde{\mu }}_{v}^{C_{2}}\left(t\right)\right\}. \end{array}$$
(b) Flow related to lower limb calculates from union the amounts of optical flow in *C*_1_ and *C*_2_ with each other in time *t*:
  (14) $$\begin{array}{c} \mu_{\text{Lower-Limb}}\left(t\right)={\tilde{\mu }}_{v}^{C_{3}\cup C_{4}}\left(t\right)=\max\left\{{\tilde{\mu }}_{v}^{C_{3}}\left(t\right),{\tilde{\mu }}_{v}^{C_{4}}\left(t\right)\right\}. \end{array}$$

*Optional.* Flow of left and right limb is calculated by considering the optical flow membership amount among *C*_1_, *C*_3_ and *C*_2_, *C*_4_, respectively:

(15) $$\begin{array}{c} \mu_{\text{Left-Limb}}\left(t\right)={\tilde{\mu }}_{v}^{C_{1}\cup C_{3}}\left(t\right)=\max\left\{{\tilde{\mu }}_{v}^{C_{1}}\left(t\right),{\tilde{\mu }}_{v}^{C_{3}}\left(t\right)\right\},\\ \mu_{\text{Right-Limb}}\left(t\right)={\tilde{\mu }}_{v}^{C_{2}\cup C_{4}}\left(t\right)=\max\left\{{\tilde{\mu }}_{v}^{C_{2}}\left(t\right),{\tilde{\mu }}_{v}^{C_{4}}\left(t\right)\right\}. \end{array}$$

This part is optional suggested but we have not used it.

(4)For the following one fuzzy IF-THEN rule, perform defuzzification.

*R1s.* If every membership function from the subject has maximum degree in membership function as compared with others, then the subject limits just some relevant action eligible for form pathway selection and selection possibility of other actions by form pathway is eliminated.

(5) Output of aforementioned membership values can be considered as belonging scores among the classes of actions which shows specific movements in human subject limbs. The biggest amount as the degree of belongs for each class will win among other amount. For example, running, jogging, and walking involve the lower limb activities whereas boxing, clapping, and waving make flow in the upper limb of human object.

### 3.5. Selecting Video Frames

**Figure 1:**
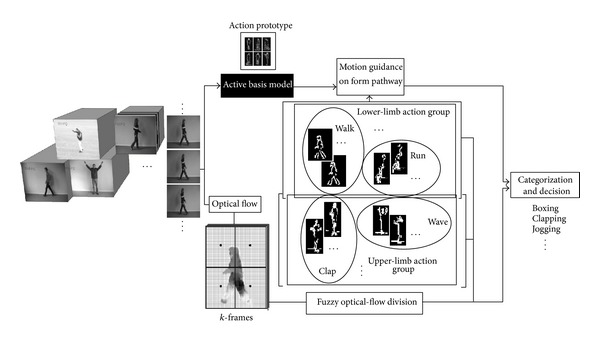
The figure reveals the flowchart of our algorithm regarding human action recognition. The flowchart presenting the hierarchical model in terms of theoretical and computation for combination of the form information and motion information is shown. A supervised Gabor based object recognition method, ABM, gives this property to have human object and computation of the form data and its combination with optical flow information, motion information, creates interaction between the pathways.

**Figure 2:**
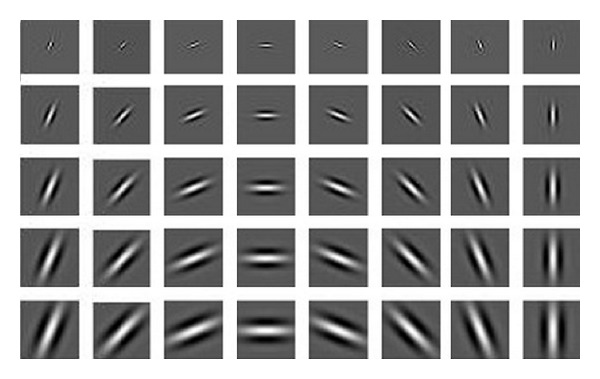
Gabor wavelets filter bank which has been used for the active basis model.

Motion analysis, video processing, and action recognition are based on frame selection for temporal order. Choosing frames based on randomization methods of temporal order can destroy the biological perception of movement [8]. Frames selection through input movie follows proposed model of form and motion pathway connection from snapshot neurons. Snapshots follow temporal order regarding configuration motion patterns of object different activity in both pathways. Proposed model uses feedforward structure for form connection (active basis function) and motion pathway (optical flow). Three frames as minimum number of the frames for snapshots will be taken from video streams following temporal order and motion information induces active basis function through feedforward joining in share sketch algorithm and makes connection appropriate (see Figure 1).

### 3.6. Relation to Existing Methods

The proposed approaches like current techniques regarding human action recognition are basically very similar to each other. In this part we will signify differences and similarities. Similar with [2, 4, 8, 22], the proposed follows original model of biological movements. The approach is made based on object recognition following hierarchical feedforward designs like [22] and specially tries to develop a model that follows neurobiological motion processing in visual cortex and basically follows [8]. Object recognition task in form pathway has been changed within the researchers work from spatiotemporal features in [22, 40] and original Gabor filter [2] for proposed approach by using active basis model. However, active basis model has basic characteristic of previous features and basically uses Gabor wavelet but it decreases matching operation. It activates limited clutters and ensures the important amounts in points of interest which falls on the person subject. The motion feature which generated through layer-wised optical flow [25] has similarity with silhouette from moving object. In our work, it is used in helping active basic model to concentrate on the object and prevent wastage of Gabor beams. Additionally, as it is previously mentioned proposed approach follows biologically inspired model [8] through parallels to visual cortex.

## 4. Evaluation and Results

To estimate the ability of the proposed approach to human action recognition, a famous human action and the largest databases, that is, the KTH human action dataset [39] and Weizmann human action recognition robustness set [31, 41], are implemented in the tests. KTH action dataset is the largest human action dataset including 598 action sequences that it comprises six types of single person actions as boxing, clapping, jogging, running, walking, and waving. These actions are performed by 25 people in different conditions: outdoors (s1), outdoors with scale variation (s2), outdoors with different clothes (s3), and indoors with lighting variation (s4). Here, using downsampling the sequences resolutions become 200 142 pixels. For our approach, we used 5 random cases (subjects) for training and making the form and motion predefined templates. As it is mentioned in the literature, KTH is a robust intrasubject variation with large set whereas the camera for taking the video during the preparation had some shacking and it makes the work with this database very difficult. Moreover, it has four scenarios which are independent, separately trained, and tested (i.e., four visually different databases, which share the same classes). Both alternatives have been run. For considering the symmetry problem of human actions, there is a mirror function for sequences along with vertical axis which can be available for testing and training sets. Here all possible overlapping of human actions within the training and testing sets has been considered (e.g., one video has 32 and 24 action frames).

### 4.1. Contribution between Motion and Form Features

**Figure 3:**
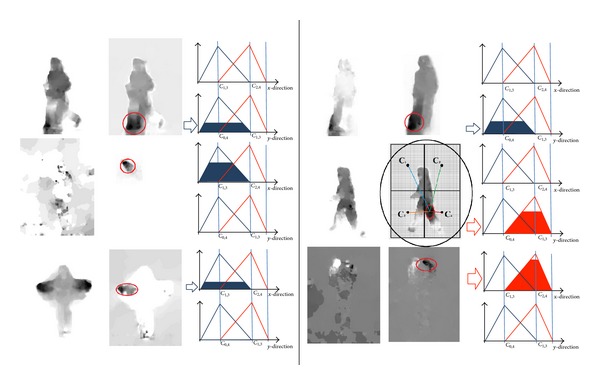
The results of dorsal processing stream obtains through optical flow [25] along with division procedures have been depicted. The resolution of divisions is designed for categorization of actions group to have additional interfere of dorsal and ventral processing streams. It can be a good representative of the interaction on MT, middle temporal of dorsal stream, and V4, ventral stream (for shape and orientation), or the MST area with inferior temporal (IT) (see more details in [32, 33]). The membership function of the action will be estimated from the position of maximum flow in the flow image. Membership values are aggregated through the proposed technique to increase the robustness. The input image of action mentioned in the figures is obtained from KTH human action recognition dataset [39].

Major strength compared with other human action recognition methods is utilizing fuzzy optical flow division for guidance of share sketch algorithm in active basis model. It combined the form and motion pathways with respect to original model. Regarding combination, a question may arise that is it necessary to combine these two pathways? and how do these two combine? The way that we have applied active basis model to form pathway and its adjustment using motion pathway information which makes the proposed method modified as compared with original model also using optical flow division guidance for active basis model is very much successful to prevention of Gabor beams wastage that it presents novelty as compared with common methods. We applied optical flow for updating active basis model point of application by evaluating the velocity of object with guidance of each optical flow division in form of fuzzy membership function (see Figure 3). It means that somehow information attained from motion pathway helps form pathway. However, combination of motion and form regularly overtakes both motion and form separately, in most of experiments conducted; combining information of these two pathways takes place in the final decision part (see [2, 8, 22]). Besides, relative feedforward structure from input data stream till final decision does not change and is similar across different datasets among two independent sets of features computed (see Figure 1 in [8] and Figure 2 in [2]). The proposed approach has been presented before [42] as the fuzzy optical flow divisions have not applied on it. Here, we have presented that with respect to the original model regarding both pathways, extracted features for each pathway can be relevant and feedforward structure has been modified and extracted features for both pathways considered having dependent information.

### 4.2. Results

Here, the biologically inspired model for human action recognition has been studied. Principally, we have described the form features attained from active basis model that it represents features form from pathway. It is also mentioned that active basis model adjusts by motion pathway information and utilizing fuzzy optical flow division regarding adjustment for increasing the accuracy of recognition. Afterward, we proceeded for application of feature selection regarding the experiments. We also prepared action prototype regarding every specific movement for human objects applying synergetic neural network. These templates are made by extracting two times prototypes from applying synergetic neural networks on train set of our human action dataset. Finally for examination of proposed approach, we applied the approach to very famous datasets which have been used for presentation of accuracy.

### 4.3. How Are Action Prototypes Created?

**Figure 4:**
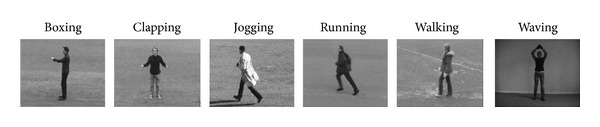
The figure depicts KTH human action dataset. To test the recognition of biological movements one of the well-known human action recognition datasets has been utilized in its performance. Here, the set represents KTH human action dataset. It is noticeable to mention that KTH dataset is one of the largest human action datasets having six various human actions in four different scenarios.

**Figure 5:**
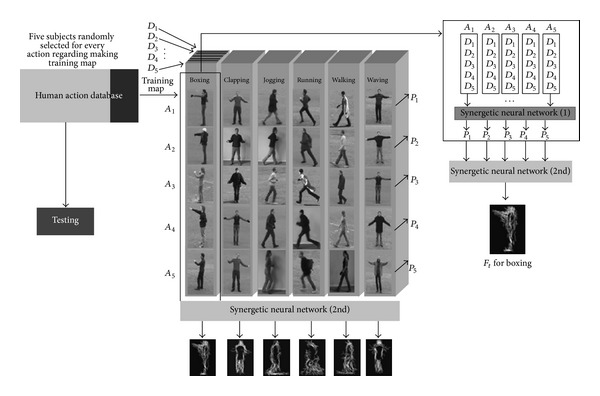
The figure shows procedure of making the action active basis templates by applying two times synergetic neural networks melting on the training map which calculates from randomly selected video frames from KTH human action database [39].

**Figure 6:**
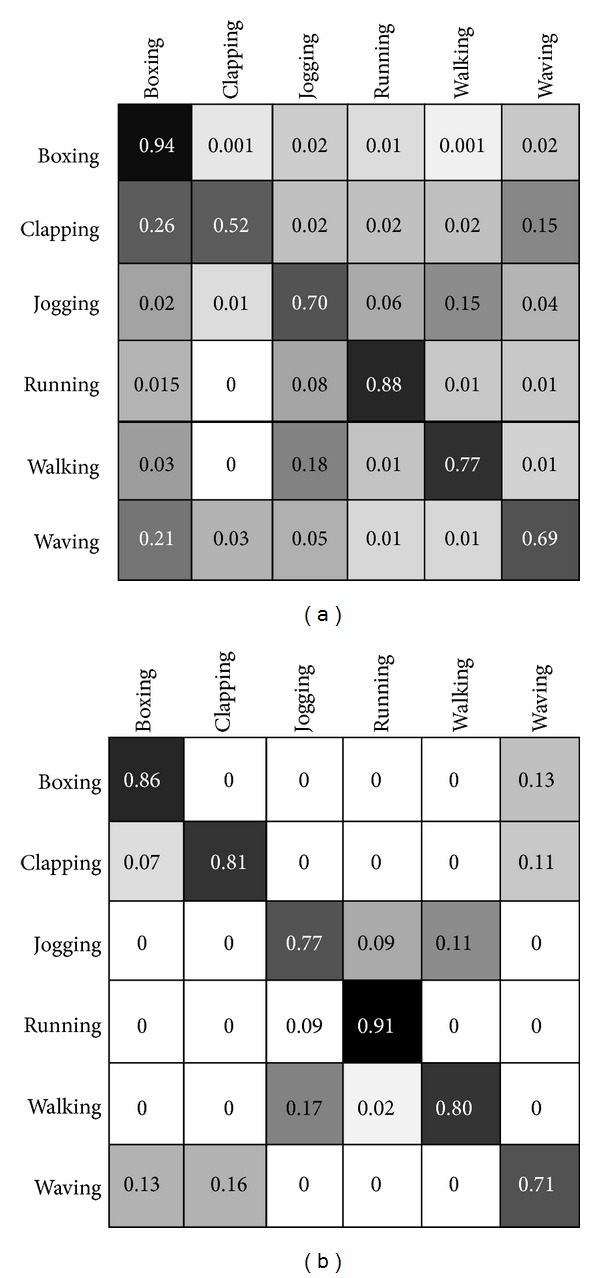
Confusion matrices SNN classifying KTH dataset obtained by adapted active basis model as combination of form and motion pathways. Confusion matrices of the proposed approach has been presented for the case of without fuzzy interference system, left matrix, and after it, right matrix which are achieved from human action movements of KTH dataset [39]. The robustness of the method after adding the fuzzy interference stabilizer is considerably increased. The wrong recognitions in the left confusion matrix have been decreased especially in case of some actions, that is, clapping. Moreover, soar of robustness helps to increase the overall accuracy and better results in classification of biological movement. The accuracy of categorizations using unbalanced SNN reached 86.46%.

As it is previously mentioned, predefined templates for each human action are obtained by applying the synergetic neural network on the human action image. For making the training map of every action, we divide every human action sequence to five primitive basic movements. One can create the whole action sequence using these five basic actions. Besides, considering the style invariance difficulties regarding diverse object in the same action, the proposed training map attains using five different subjects from targeted human action databases. For easing the explanation, we consider five snippets in different actions *A*_1_ − *A*_5_ and each subject from targeted database *D*_1_ − *D*_5_. First, synergetic neural network applies to *A*_1_ in *D*_1_ − *D*_5_  and outcome shows by *P*_1_ as first prototype obtains from first action snippet. The number of prototypes will be completed by applying the synergetic neural network and calculating the residual prototypes that they have called *P*_1_ − *P*_5_. Calculated prototype images considering style invariance represent the one action within five snapshots. Afterward, these prototypes melt together using second time synergetic neural network for attaining the final prototypes where each of them represents the specific action within different action snippets and considering style invariance property. Let *F*_*t*_ represents outcome of melting *P*_1_ − *P*_5_ in specified action. The final prototypes along with the method which they calculated are depicted in Figure 4. The confusion metric reveal the significant of using fuzzy inference system inside the model of the biological movement. The difference among these two confusion matrixes is very big and it can prove the advantage of using fuzzy optical flow division for this context. Furthermore, Table 1 reveals a comparison of our method with other methods in terms of recognition of accuracy in which the accuracy result indicates that the accuracy of proposed technique is relatively comparable with state-of-art by considering that there are somehow two categories using not very similar paradigms, which cannot be straightly compared. Here, the experimental result of proposed approach is presented. As KTH human action database [39] has been used for benchmarking the accuracy of consistency with set of experiments used in [2, 22, 27, 43, 44], we made a set of our training map and test set for proposed technique on the entire dataset, in which the mixture of four scenarios videos was together. The dataset split into a set of training maps with five randomly selected subjects and a test part by residual subjects. Afterward, we measured the average performance over five random splits. The training map dataset was very small and comprised five videos frames snippets randomly obtained from the mixture dataset.  Figure 5 presents classification confusion matrices for KTH dataset. Rows of confusion matrix represent the corresponding classification results, where each column signifies the instances to be classified. In proposed approach, the highest confusion happens among walking, jogging, and running. Discriminating these actions is difficult as the performance of actions by some subjects has similarity (see Figure 6). Also, another misclassification happens principally between similar classes, like previous confusion or hand clapping, hand, and waving (see confusion matrices in Figure 5).

### 4.4. Does the Fuzzy Optical Flow Division Help to Have Better Accuracy?

As it is mentioned in the previous parts of the paper, regarding obtaining action prototypes through synergetic neural network during one whole action frames. This method can give a good abstract from the action video but it has a problem [42] which decreases our accuracy due to cluttered areas in the action prototypes. Following this problem, there was similarity among the matched image frames which is the cause of disparity in accuracy and it is very clearly revealed in confusion matrix (see Figure 5 upper confusion matrix). Presented approach has considered 0.1 second for, 2 for, *k* = 3 means three frame time considered as dependency of prevent membership function value and attained from training on our training set. During our experiment, we just have applied upper and lower limb membership functions and left and right limb functions can be suggested for more complex actions. After applying fuzzy optical flow division, disparity dramatically diminished. Confusion matrix after applying this method has been shown in Figure 5 (second confusion matrix).

### 4.5. Related Work

Human action recognition tasks are generally categorized as two separated classes. First class prefers to track the part of image which is object (human) exists [45]. Mentioned groups of techniques might not be useful in less articulated objects. However, they are considered as successful approaches. The other popular class is addressed on low resolution videos or high locally resolution images [46] or by using spatiotemporal features [4, 40]. As it has previously been discussed regarding neurobiologically inspired model for analysis of movement in dorsal stream visual cortex and psychological and physiological information, our proposed approach is categorized as second group of methods. Previous method [8] has constant translation lack and a limited handcrafted features dictionary in intermediate periods [47], Jhuang et al. (2007) [22] and Schindler et al. [2].

### 4.6. Discussion

**Figure 7:**
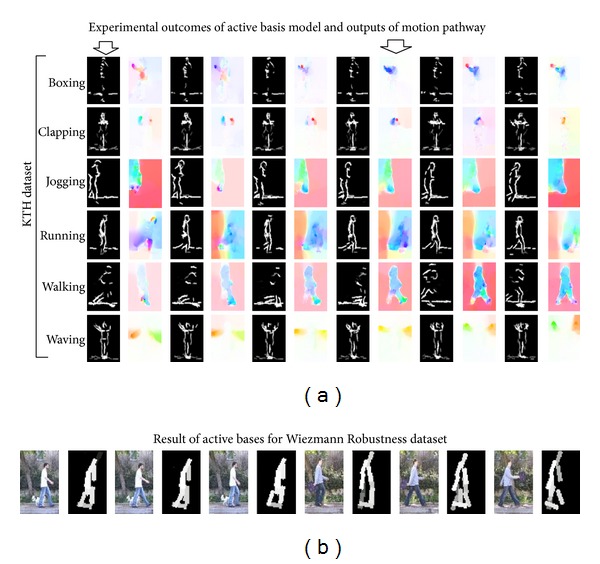
Simulation results for simple biological movement paradigm based on ABM [24] in the ventral processing stream and optical flow [25] in dorsal stream are shown. Each row within the panel reveals the response of ABM during the episode as well as flow generated for every different action. The set of biological movements that belongs to the biological movements is from KTH dataset [39]. (a) The simulation results of the different actions of KTH dataset along with results of optical flow simulation; (b) the figure depicts some results of Weizmann robustness dataset. It reveals an increase in the robustness of the proposed approach due to utilization of ABM [24] in the ventral stream.

In the previous sections, we revealed the biologically inspired model regarding human action recognition using active basis model and fuzzy optical flow division. Here, we discuss advantages and limitations of our models, in addition to comparative explanation plus relationship of the proposed approach with existing models (see [2, 8, 48, 49]). We demonstrated how to apply a supervised learning Gabor based method which successfully has been utilized before for the task of object recognition previously [24] for the form pathway. As the form pathway is considered for ventral stream representation, it has a task of object recognition biologically. Active basis model can learn the human object considering the prototypes and is able to find it within frames. Such a property is very much desirable for visual system representation in the model; however, this part has been done by Gabor wavelet in the previous models [8, 48, 49] and similar works [2]. It could follow the involving encode object shape [32]. The object shape concern in the form pathway and ventral stream has been properly considered based on training stage and human prototypes. Using active basis model is somehow considered as the Gabor action stimulus for pin down form processing at two levels local information about limb angle from Gabor orientations and global body structure signaled by the spatial arrangement of Gabor paths. On the other hand, using optical flow for extraction of motion information has followed the second attribute and involves filtering by direction selection sensors and its integration for solving the famous aperture problem. Motion information presents both types of motion signals local velocity and joint motion trajectories will be signals to form path by guiding SSA in active basis model [50] as a good representation of cross-connection between V4 and MT. It follows the predominant view of form and motion processing in the human visual system which assumes that these two attributes are handled by independent and separate modules ([2, 8, 48, 49]). It has been recognized that form signal information can influence processing of motion more extensively than previously thought (see [32]) and the proposed approach considers direct effect on motion information on the form processing. The connectivity within the visual system is characterized by cross-connections in respect of parallel feedforward connection ([33, 51, 52]). Using optical flow division technique provides connection and interaction of bottom-up and top-down processing among brain regions along the dual computational streams. Also dorsal stream is assumed to preform complementary spatial computation (where) and ventral stream for performing object recognition (what) in the cortical areas V1, V2, V4, and IT (inferotemporal cortex) along with current evidence in opposition to a complete segregation of where and what information in the brain of macaque (see [52, 53]) representing that information about position and size of objects is also represented in inferotemporal cortex of macaques as top layer of ventral stream. However, in the proposed approach an early isolation of spatial configuration and identity into divided processing pathways need heavy computation in hardware. But having low resolution optical flow divisions (four divided parts) could be a good parameter for diminishing this computational load. The correctly classified sequences are reported as highest results in literature. To place proposed technique in this context, we have presented it with state-of-the-art. Our method similar with other methods which is frame-based runs for all frames of action sequences. Then the individual labels obtained from training map are simply compared to a sequence label through majority voting (it is like a bag-of-frames model and like [2, 40, 48]). The comparison with state-of-the-art has been done and it is revealed in Table 1. It accuracy considering comparing with other methods indicates relative compatibility for proposed approach. In terms of contribution among motion and form features, we can mention applying active basis model which modified the form pathway itself considering it as a Gabor based model and its ability for learning the object increases the robustness of the system which is tested using Weizmann robustness dataset. Moreover, optical flow guidance for SSA as cross-connection among the dual computational streams plus prevention of application of Gabor beams for nontargeted objects is depicted in Figure 7. Considering fuzzy optical flow division keeps system robust, the proposed method can be categorized as an improvement in this field. However, the natural question (see [2, 48]) regarding whether this combination is necessary or how to improve it is still there and researchers are still trying to expand the model and make it more accurate. We have performed experiments following presented method, in which we have modified form pathway and made it combined with motion path and made a relation for these two independent feature sets and its connection which revealed promising results.

## 5. Conclusion

In this paper, a human action recognition method has been proposed; this method is based on interrelevant calculated motion and form information followed the biologically inspired system. The active basis model applied for generating the form information and optical flow guides the share sketch algorithm regarding better concentration on human object in the video frames and it can represent cross-connection of V4 and MT in brain [49]. Synergetic neural network is used twice on training set finding action prototypes for each action. The approach has been tested for KTH and robustness-Weizmann human action dataset and experimental assessment of the proposed technique has shown promising results which was relatively comparable with state-of-the-art methods and benefit of proposed cross connection into the feed-forward method on the biological movement. Also it had good performance on different datasets and its training is done by less computational load regarding final action prototypes learning and reasonable computational cost. As a limitation of the proposed approach, it presently does not have mechanisms for invariance against rotation and viewpoint changes whereas it is capable to put mechanism regarding multiscale. Also active basis model is very sensitive algorithm which needs attention on while it is training. As open questions, motion sequences consistently represent recognition of video stream is done in which frame. How much these two pathways clearly follow the biologically inspired movement of mammalian brain. Future work will extend the proposed approach better integration of form and motion information in pathways. Another extension is to find better techniques to not using action prototypes regarding human action recognition.

## Figures and Tables

**Table 1:** The recognition results of proposed method has presented along with comparison between previous human action recognition method (Bio or non bio-inspired) on KTH human action dataset.

Methods	Accuracy (%)	Years
Wang et al. [40]	71.72	2004
Niebles et al. [43]	83.33	2006
Jhuang et al. [22]	91.79	2008
Schindler and Van Gool [2]	92.79	2009
Wang and Mori [27]	91.29	2009
	U-SFA: 86.67	
Zhang and Tao [44]	S-SFA: 86.40	2012
	D-SFA: 89.33	
	SD-SFA: 93.87	
Proposed Method	86.46	2013

## Appendix

In this part, we are presenting an example of proposed approach in fuzzy optical flow division part which is shown and proving the performance of time dependency in suggested fuzzy inference system for proposed application. Suppose that system in steady-state situation at the time of *t*_0_, *β* = *β*′ is equal to 2 and *τ* = 0.1 *s*. Consider

(A.1) $$\begin{array}{c} \mu_{v}^{C_{1}}\left(t\right)=0.6,\;\mu_{v}^{C_{2}}\left(t\right)=0.2,\\ \mu_{v}^{C_{3}}\left(t\right)=0.4,\;\mu_{v}^{C_{4}}\left(t\right)=0.5. \end{array}$$

Cell *C*_1_ is winner and represents the highest optical flow. So, we will have timing influence by

(A.2) $$\begin{array}{c} k=0,\;\;t=t_{0},\;\;{\tilde{\mu }}_{v}^{C_{z}}\left(t-\tau \right)=0, \end{array}$$

(A.3) $$\begin{array}{c} {\tilde{\mu }}_{v}^{C_{z}}\left(t_{0}\right)=0.6, \end{array}$$

For *k* = 1, *t*′ = *t*_0_ + *τ*,

(A.4) $$\begin{array}{c} \mu_{v}^{C_{1}}\left(t^{\prime }\right)=0.59,\;\;\mu_{v}^{C_{2}}\left(t^{\prime }\right)=0.3,\\ \mu_{v}^{C_{3}}\left(t^{\prime }\right)=0.1,\;\;\mu_{v}^{C_{4}}\left(t^{\prime }\right)=0.5. \end{array}$$

As the highest cell did not change (condition of $\mu_{v}^{C_{j}}(t^{\prime })\leq {\tilde{\mu }}_{v}^{C_{i}}(t_{0})$ ), having memory coefficient function (*η*_*v*_^*C*_1_^(*t*)) chosen 1/(*k* + *β*) and considering ${\tilde{\mu }}_{v}^{C_{1}}(t-\tau )=0.6$, we will have

(A.5) $$\begin{array}{c} {\tilde{\mu }}_{v}^{C_{1}}\left(t^{\prime }\right)=0.6+\frac{1}{1+2}\left(1-0.6\right)=0.73. \end{array}$$

Also having *N* = 3, we will have

(A.6) $$\begin{array}{c} k=2,\;\;t^{\prime \prime }=t_{0}+2\tau ;\\ \mu_{v}^{C_{1}}\left(t^{\prime \prime }\right)=0.7,\;\;\mu_{v}^{C_{2}}\left(t^{\prime \prime }\right)=0.5,\\ \mu_{v}^{C_{3}}\left(t^{\prime \prime }\right)=0.25,\;\;\mu_{v}^{C_{4}}\left(t^{\prime \prime }\right)=0.45,\\ k=3,\;\;t^{\prime \prime \prime }=t_{0}+3\tau ;\\ \mu_{v}^{C_{1}}\left(t^{\prime \prime \prime }\right)=0.8,\;\;\mu_{v}^{C_{2}}\left(t^{\prime \prime \prime }\right)=0.1,\\ \mu_{v}^{C_{3}}\left(t^{\prime \prime \prime }\right)=0.4,\;\;\mu_{v}^{C_{4}}\left(t^{\prime \prime \prime }\right)=0.6,\;\;{\tilde{\mu }}_{v}^{C_{1}}\left(t^{\prime }\right)=0.73,\\ {\tilde{\mu }}_{v}^{C_{1}}\left(t^{\prime \prime \prime }\right)=0.73+\frac{1}{2+2}0.26=0.796. \end{array}$$

After this by having maximum optical flow division for first cell, *k* will be reset to zero and this cycle will be repeated by choosing the maximum membership function for next step. But if suddenly after *k* = 0, some other cell gets highest optical flow, we will have

(A.7) $$\begin{array}{c} k=3;\;\;t^{\prime }=t_{0}+\tau ,\\ \mu_{v}^{C_{1}}\left(t^{\prime }\right)=0.4,\;\;\mu_{v}^{C_{2}}\left(t^{\prime }\right)=0.3,\\ \mu_{v}^{C_{3}}\left(t^{\prime }\right)=0.6,\;\;\mu_{v}^{C_{4}}\left(t^{\prime }\right)=0.5. \end{array}$$

*C*_3_ won having the highest optical flow in its division, so cellular changing occurred but the result of the membership function is changing gradually and 1/(*k* + *β*′) will be chosen for memory coefficient function and we will have

(A.8) $$\begin{array}{c} {\tilde{\mu }}_{v}^{C_{1}}\left(t-\tau \right)=0.73,\\ {\tilde{\mu }}_{v}^{C_{1}}\left(t^{\prime }\right)=0.73-\frac{1}{1+2}0.26=0.64. \end{array}$$

As ${\tilde{\mu }}_{v}^{C_{1}}(t^{\prime })>{\tilde{\mu }}_{v}^{C_{3}}(t^{\prime })$ still first cell wins

(A.9) $$\begin{array}{c} k=2;\;\;t^{\prime \prime }=t_{0}+2\tau ,\\ \mu_{v}^{C_{1}}\left(t^{\prime \prime }\right)=0.2,\;\;\mu_{v}^{C_{2}}\left(t^{\prime \prime }\right)=0.5,\\ \mu_{v}^{C_{3}}\left(t^{\prime \prime }\right)=0.68,\;\;\mu_{v}^{C_{4}}\left(t^{\prime \prime }\right)=0.6,\\ {\tilde{\mu }}_{v}^{C_{1}}\left(t^{\prime }\right)=0.64,\;\;{\tilde{\mu }}_{v}^{C_{1}}\left(t^{\prime \prime }\right)=0.64-\frac{1}{2+2}0.36=0.55. \end{array}$$

And by considering the time when first cell won this amount will be increased but if the change continues third cell will win and we will have reset in *k* and final choosing of the cell. Also, if the amount of membership function changes in *k* = 2 and back to the previous cell, we will have

(A.10) $$\begin{array}{c} k=2;\;\;t^{\prime \prime }=t_{0}+2\tau ,\\ \mu_{v}^{C_{1}}\left(t^{\prime \prime }\right)=0.7,\;\;\mu_{v}^{C_{2}}\left(t^{\prime \prime }\right)=0.5,\\ \mu_{v}^{C_{3}}\left(t^{\prime \prime }\right)=0.4,\;\;\mu_{v}^{C_{4}}\left(t^{\prime \prime }\right)=0.6,\\ {\tilde{\mu }}_{v}^{C_{1}}\left(t^{\prime }\right)=0.64,\;\;{\tilde{\mu }}_{v}^{C_{1}}\left(t^{\prime \prime }\right)=0.64-\frac{1}{2+2}0.36=0.73. \end{array}$$

And there will be no sudden changes on final decision. It means first cell will be constantly selected. Also by selecting the suitable *β*′ the system will have minor sensitivity regarding cellular varying. But it is abundantly related to the application and video condition.

## References

[1] Santofimia MJ, Martinez-del-Rincon J, Nebel JC Episodic reasoning for vision-based human action recognition.

[2] Schindler K, Van Gool L Action Snippets: how many frames does human action recognition require?.

[3] Hogg T, Rees D, Talhami H Three-dimensional pose from two-dimensional images: a novel approach using synergetic networks.

[4] Efros AA, Berg AC, Mori G, Malik J Recognizing action at a distance.

[5] Daugman JG (1980). Two-dimensional spectral analysis of cortical receptive field profiles. *Vision Research*.

[6] Olshausen BA, Field DJ (1996). Emergence of simple-cell receptive field properties by learning a sparse code for natural images. *Nature*.

[7] Riesenhuber M, Poggio T (2002). Neural mechanisms of object recognition. *Current Opinion in Neurobiology*.

[8] Giese MA, Poggio T (2003). Neural mechanisms for the recognition of biological movements. *Nature Reviews Neuroscience*.

[9] Thorpe S, Fize D, Marlot C (1996). Speed of processing in the human visual system. *Nature*.

[10] Oram MW, Perrett DI (1996). Integration of form and motion in the anterior superior temporal polysensory area (STPa) of the macaque monkey. *Journal of Neurophysiology*.

[11] Johansson G (1976). Spatio-temporal differentiation and integration in visual motion perception—an experimental and theoretical analysis of calculus-like functions in visual data processing. *Psychological Research*.

[12] Riesenhuber M, Poggio T (1999). Hierarchical models of object recognition in cortex. *Nature Neuroscience*.

[13] Riesenhuber M, Poggio T (2000). Models of object recognition. *Nature Neuroscience*.

[14] Kourtzi Z, Kanwisher N (2000). Activation in human MT/MST by static images with implied motion. *Journal of Cognitive Neuroscience*.

[15] Saleem KS, Suzuki W, Tanaka K, Hashikawa T (2000). Connections between anterior inferotemporal cortex and superior temporal sulcus regions in the macaque monkey. *Journal of Neuroscience*.

[16] Giese MA, Vaina LM (2001). Pathways in the analysis of biological motion: computational model and fMRI results. *Perception*.

[17] Jones JP, Palmer LA (1987). An evaluation of the two-dimensional Gabor filter model of simple receptive fields in cat striate cortex. *Journal of Neurophysiology*.

[18] Dow BM, Snyder AZ, Vautin RG, Bauer R (1981). Magnification factor and receptive field size in foveal striate cortex of the monkey. *Experimental Brain Research*.

[19] Lampl I, Ferster D, Poggio T, Riesenhuber M (2004). Intracellular measurements of spatial integration and the MAX operation in complex cells of the cat primary visual cortex. *Journal of Neurophysiology*.

[20] Gawne TJ, Martin JM (2002). Responses of primate visual cortical V4 neurons to simultaneously presented stimuli. *Journal of Neurophysiology*.

[21] Logothetis NK, Pauls J, Poggio T (1995). Shape representation in the inferior temporal cortex of monkeys. *Current Biology*.

[22] Jhuang H, Serre T, Wolf L, Poggio T A biologically inspired system for action recognition.

[23] Eifuku S, Wurtz RH (1998). Response to motion in extrastriate area MSTI: center-surround interactions. *Journal of Neurophysiology*.

[24] Wu YN, Si Z, Gong H, Zhu S-C (2010). Learning active basis model for object detection and recognition. *International Journal of Computer Vision*.

[25] Liu C (2009). *Beyond pixels: exploring new representations and applications for motion analysis [Ph.D. thesis]*.

[26] Tanha J, Van Someren M, Afsarmanesh H An AdaBoost algorithm for multiclass semi-supervised learning.

[27] Wang Y, Mori G (2009). Human action recognition by semilatent topic models. *IEEE Transactions on Pattern Analysis and Machine Intelligence*.

[28] Alvarez L, Deriche R, Papadopoulo T, Sánchez J (2007). Symmetrical dense optical flow estimation with occlusions detection. *International Journal of Computer Vision*.

[29] Brox T, Bruhn A, Papenberg N, Weickert J High accuracy optical flow estimation based on a theory for warping.

[30] Bruhn A, Weickert J, Schnörr C (2005). Lucas/Kanade meets Horn/Schunck: combining local and global optic flow methods. *International Journal of Computer Vision*.

[31] Blank M, Gorelick L, Shechtman E, Irani M, Basri R Actions as space-time shapes.

[32] Mather G, Pavan A, Marotti RB, Campana G, Casco C (2013). Interactions between motion and form processing in the human visual system. *Frontiers in Computational Neuroscience*.

[33] Cloutman LL (2013). Interaction between dorsal and ventral processing streams: where, when and how?. *Brain and Language*.

[34] Lee GC, Loo CK Facial pose estimation using modified synergetic computer.

[35] Zadeh LA (1965). Fuzzy sets. *Information and Control*.

[36] Kumar S (2004). *Neural Networks: A Classroom Approach*.

[37] Leotamonphong J, Fang S (1999). An efficient solution procedure for fuzzy relation equations with max product composition. *IEEE Transactions on Fuzzy Systems*.

[38] Chen B, Liu X (2005). Delay-dependent robust H∞ control for T-S fuzzy systems with time delay. *IEEE Transactions on Fuzzy Systems*.

[39] Schüldt C, Laptev I, Caputo B Recognizing human actions: a local SVM approach.

[40] Wang B, Liu Y, Wang W, Xu W, Zhang M (2013). Multi-scale locality-constrained spatiotemporal coding for local feature based human action recognition. *The Scientific World Journal*.

[41] Gorelick L, Blank M, Shechtman E, Irani M, Basri R (2007). Actions as space-time shapes. *IEEE Transactions on Pattern Analysis and Machine Intelligence*.

[42] Yousefi B, Loo CK, Memariani A Biological inspired human action recognition.

[43] Niebles JC, Wang H, Fei-Fei L (2008). Unsupervised learning of human action categories using spatial-temporal words. *International Journal of Computer Vision*.

[44] Zhang Z, Tao D (2012). Slow feature analysis for human action recognition. *IEEE Transactions on Pattern Analysis and Machine Intelligence*.

[45] Bregler C, Malik J, Pullen K (2004). Twist based acquisition and tracking of animal and human kinematics. *International Journal of Computer Vision*.

[46] Dollár P, Rabaud V, Cottrell G, Belongie S Behavior recognition via sparse spatio-temporal features.

[47] Fanti C, Zelnik-Manor L, Perona P Hybrid models for human motion recognition.

[48] Danafar S, Gretton A, Schmidhuber J Characteristic kernels on structured domains excel in robotics and human action recognition.

[49] Kruger N, Janssen P, Kalkan S (2013). Deep hierarchies in the primate visual cortex: what can we learn for computer vision?. *IEEE Transactions on Pattern Analysis and Machine Intelligence*.

[50] Thurman SM (2013). Complex interactions between spatial, orientation, and motion cues for biological motion perception across visual space. *Journal of Vision*.

[51] Distler C, Boussaoud D, Desimone R, Ungerleider LG (1993). Cortical connections of inferior temporal area TEO in macaque monkeys. *Journal of Comparative Neurology*.

[52] Hung CP, Kreiman G, Poggio T, DiCarlo JJ (2005). Fast readout of object identity from macaque inferior temporal cortex. *Science*.

[53] Lehky SR, Peng X, McAdams CJ, Sereno AB (2008). Spatial modulation of primate inferotemporal responses by eye position. *PLoS ONE*.

